# Genome sequences of two *Escherichia coli* bacteriophages isolated from activated sludge in domestic wastewater treatment plants

**DOI:** 10.1128/mra.00285-25

**Published:** 2025-08-11

**Authors:** Tadashi Nittami, Kaho Motoyama, Minami Sasaki, Yugo Fujii, Steven Batinovic, Mwila Kabwe, Joseph Tucci

**Affiliations:** 1 Division of Materials Science and Chemical Engineering, Yokohama National University, Yokohama, Kanagawa, Japan; 2 Department of Chemistry and Life Science, Yokohama National University, Yokohama, Kanagawa, Japan; 3 Holsworth Biomedical Research Centre, Department of Rural Clinical Sciences, La Trobe Rural Health School, La Trobe University 2080, Bendigo, Victoria, Australia; Katholieke Universiteit Leuven 26657, Leuven, Belgium

**Keywords:** wastewater treatment, activated sludge

## Abstract

Two bacteriophages infecting *Escherichia coli* were isolated from activated sludge samples collected from two different domestic wastewater treatment plants in Japan. One phage is related to *Kayfunavirus* phages and the other to *Kuravirus* phages. We report the complete genome sequences of these isolates.

## ANNOUNCEMENT

Public health concerns regarding antibiotic resistance in pathogens such as *Escherichia coli* have prompted the search for alternative methods to control them. Bacteriophages (phages) have received increasing attention as potential alternatives (1–3). We isolated two *E. coli* phages (KM01 and KM02); each phage was isolated from an activated sludge sample collected at two different domestic wastewater treatment plants in Yokohama, Japan. The phages were isolated on host *E. coli* strain K12 (JCM20135—from feces of a diphtheria patient). Phage isolation and genomic DNA extraction, using phenol-chloroform with zinc chloride precipitation, were conducted as previously described (4, 5). Default parameters were used for all software tools, unless otherwise specified.

DNA libraries were constructed without fragmentation or size selection using Ligation Sequencing Kit 14 (SQK-LSK114) and loaded onto R10.4.1 flow cells (FLO-MIN114) for sequencing with MinION Mk1B (Oxford Nanopore, Tokyo, Japan). Simplex and duplex basecalling used Guppy v.6.4.2 in super accurate mode. Read filtering was performed with Filtlong v.0.2.1 (https://github.com/rrwick/Filtlong). Each read had mean qualities of Q19.6 and 16.2 and mean lengths of 25.2 kb and 8.18 kb, respectively, as evaluated by NanoPlot v.1.40.0 (6). Trimming of reads was performed with Trim Galore v.0.6.7 (https://github.com/FelixKrueger/TrimGalore). The assembly process used Flye v.2.9.1 (7) in high-quality mode. Post-assembly polishing was by Medaka v.1.7.2 (https://github.com/nanoporetech/medaka) without corrections to assembly. Final assembly was screened against Y-adapter top and bottom sequences, to remove remaining Nanopore adapter sequences from ends.

To identify direct terminal repeats, we filtered the assembly using PhageTerm v.1.0.12 (8) and determined genuine genome ends. For annotation, we imported assemblies into Geneious Prime v.2025.0.3 (https://www.geneious.com/), searching for Open Reading Frames (ORFs) with Glimmer3 (9) using start codons GTG, ATG, and TTG. Gene prediction was manually performed using the NCBI Protein Blast (BLASTp) search. Table 1 shows features of genome characteristics. Characteristics of KM01 are similar to the existing *Kuravirus*-like group of phages (4), including genome size, number of ORFs (124), number of tRNA/tmRNA (0), and G + C content (42.2%; lower than host *E. coli* strain K12 [50.5%]) (https://www.ncbi.nlm.nih.gov/datasets/genome/GCF_002087985.1/). Protein similarity analysis (ViPTree) (10) indicated that KM01 belonged to subfamily Gordonclarkvirinae and genus *Kuravirus*. The NCBI genome BLAST also showed that this displayed similarity to *Escherichia* phage *Kuravirus* phiEc_1 (LC768462), with 98.1% sequence identity (95% query coverage). Phage KM02 has a smaller genome size with fewer ORFs and no tRNA/tmRNA, but its G + C content (49.7%) was higher than that of KM01. The ViPTree indicated that KM02 belonged to subfamily Studiervirinae and genus *Kayfunavirus*. NCBI genome BLAST showed the sequence displayed 95.7% sequence identity (84% query coverage) to *Kayfunavirus* MirsaeidiM_Esher2 (PQ464628) *Escherichia* phage. KM02 exhibited low query coverage against existing phages, indicating the diversity of *E. coli* phages.

**TABLE 1:** Genometrics of *E. coli* phages

Phage	Genome size (nt)	No. of ORFs	No. of tRNA /tmRNA	G + C (%)	Mean coverage (×)	Closest relative	Query coverage (%)	Identity (%)	GenBank accession
KM01	75,964	124	0	42.2	91	*Kuravirus*, *Escherichia* phage phiEc_1 (Acc. LC768462)	95	98.1	PV018827
KM02	40,673	53	0	49.7	95	*Kayfunavirus*, *Escherichia* phage MirsaeidiM_Esher2 (Acc. PQ464628)	84	95.7	PQ179042

## Data Availability

The genome sequences of the three *Escherichia* phages listed in Table 1 are available in NCBI GenBank under the accession numbers PV018827 and PQ179042. Raw sequence data are available under Sequence Read Archive (SRA) accession numbers SRR33209174 and SRR33223178.
